# Fix Your Eyes in the Space You Could Reach: Neurons in the Macaque Medial Parietal Cortex Prefer Gaze Positions in Peripersonal Space

**DOI:** 10.1371/journal.pone.0023335

**Published:** 2011-08-17

**Authors:** Kostas Hadjidimitrakis, Rossella Breveglieri, Giacomo Placenti, Annalisa Bosco, Silvio P. Sabatini, Patrizia Fattori

**Affiliations:** 1 Department of Human and General Physiology, University of Bologna, Bologna, Italy; 2 Department of Biophysical and Electronic Engineering, University of Genova, Genova, Italy; The University of Western Ontario, Canada

## Abstract

Interacting in the peripersonal space requires coordinated arm and eye movements to visual targets in depth. In primates, the medial posterior parietal cortex (PPC) represents a crucial node in the process of visual-to-motor signal transformations. The medial PPC area V6A is a key region engaged in the control of these processes because it jointly processes visual information, eye position and arm movement related signals. However, to date, there is no evidence in the medial PPC of spatial encoding in three dimensions. Here, using single neuron recordings in behaving macaques, we studied the neural signals related to binocular eye position in a task that required the monkeys to perform saccades and fixate targets at different locations in peripersonal and extrapersonal space. A significant proportion of neurons were modulated by both gaze direction and depth, i.e., by the location of the foveated target in 3D space. The population activity of these neurons displayed a strong preference for peripersonal space in a time interval around the saccade that preceded fixation and during fixation as well. This preference for targets within reaching distance during both target capturing and fixation suggests that binocular eye position signals are implemented functionally in V6A to support its role in reaching and grasping.

## Introduction

Primate lifestyle requires frequent relocations in space and coordinated movements of the eyes and hands to interact with objects. For this behavior, a reliable visual percept of three-dimensional (3D) space needs to be constructed and integrated in a movement plan involving several effectors. The posterior parietal cortex (PPC) plays a pivotal role in these processes. Humans with lesions in PPC showed deficits in perceiving the spatial relationship between objects and their own body, and performed inaccurate reaching movements [1], [2], [3]. In addition, damages to PPC were shown to affect specifically the depth component of visually-guided reaching movements [4], [5]. Psychophysical studies suggested that, in order to reach objects in depth, information about the vergence angle is critical [6], [7]. Vergence has been tightly related to the fixation distance [8], which, together with the retinal disparity signal that specifies the distance of an object from the fixation plane, have been proposed to encode object location in 3D space [9].

Several neurophysiological studies in macaques reported PPC neurons with responses related to the fixation distance (also termed fixation depth). Neurons with selectivity for the depth of fixation have been first described in area 7a [10]. In area LIP, saccadic neurons were found to have response fields tuned in depth [11], [12]. More recently, a study of the parietal reach region (PRR), a functionally defined area that probably corresponds to MIP, demonstrated that both the disparity and the vergence of the target modulated the responses of neurons involved in planning reaching movements [13]. Modulation of activity by vergence eye movements and tonic vergence signals has been also observed in frontal and temporal cortical regions [14], [15], and even in the primary visual cortex [16] and in area V4 [17].

It has also been argued that the dorsal pathway is particularly involved in the processing of visual input from the lower visual field where the hands are usually located [18]. Moreover, vergence eye movements were shown to occur most frequently in the near space, corresponding to arm's length, as they are needed for accurate reaching [19]. Interestingly, in the medial PPC area V6A, located in the anterior bank of the parieto-occipital sulcus, a prevalence of visual neurons with receptive fields that mostly represent the lower visual field has been demonstrated [20], [21]. In addition, many V6A neurons were found to be influenced by gaze position in a frontal, two-dimensional (2D) space [22], [23]. V6A has also been shown to receive somatosensory input from the upper limbs [24], and to contain arm movement-related neurons that encoded the direction of reaches [25] as well as more distal aspects of prehension, such as hand orientation [26] and grip formation [27].

Here, we studied the activity of V6A neurons when gaze is directed towards the near and lower space, as it occurs naturally during object grasping and manipulation, and when gaze is directed towards the far space. Given that vergence information is needed for interacting in peripersonal space, and given also that V6A is involved in the neural encoding of prehension [28], we investigated whether vergence information, necessary for encoding depth, is processed in this cortical area. To test this, we used an experimental configuration similar to that used in human studies [4], [5], [29], [30] in which fixation targets were placed on a horizontal board below eye level. Our hypothesis was that cells in V6A would show stronger responses for targets presented in the reachable space. We found that neurons in V6A a) respond during the shifts of gaze performed in 3D space, and b) are tonically modulated by 3D eye position. We report a preferential representation of near and downward gaze positions that span the manual workspace, a fact that well agrees with the suggested role of V6A in reaching and grasping [25], [26], [27].

## Results

We recorded neuronal activity in V6A and identified 184 well isolated, stable cells (65 from monkey M1, 53 from the right and 12 from the left hemisphere; 119 from monkey M2; 55 from the right and 64 from the left hemisphere).

**Figure 1:**
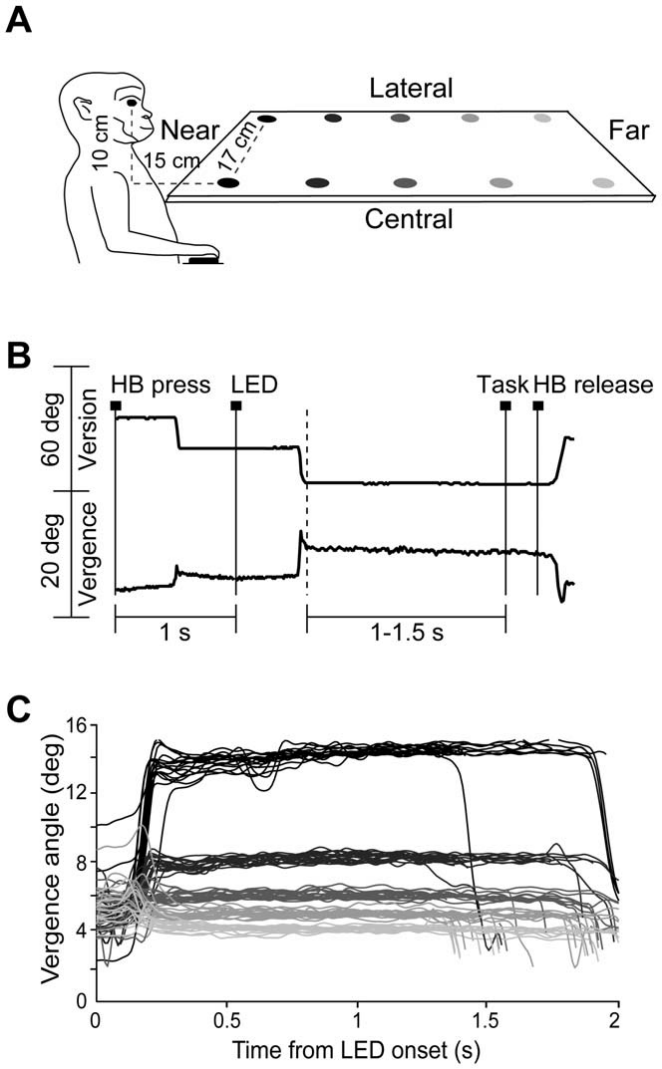
Experimental setup and task sequence. (A): Scheme of the set up used for the fixation in depth task. Eye movements were performed in darkness towards one of the ten LEDs of a horizontal panel. (B): Time course of the task. A typical example of the version (top) and vergence (bottom) traces during a single trial is shown. Long vertical continuous lines indicate the occurrence of events during the task. From left to right markers show: trial's start (HB press), target appearance (LED light on green), end of fixation (Task, green to red change of the LED) and trial end (HB release). Long vertical dashed line indicates the end of the saccade. (C): Calibrated vergence eye position signal (in degrees) during fixation of the LEDs of the central row (different tones of grey), in an example recording. Vergence angle is maintained for the whole required fixation (1 s–1.5 s) and is clearly distinguishable among the different targets.

The animals were required to fixate on ten LED targets located in different positions in the 3D space (Fig. 1A). Targets were arranged in two configurations. In the first one, they were evenly distributed along two rows, one located centrally, in the mid-sagittal plane, and the other located contralateral to the recording site, in a parasagittal plane (Fig. 1A). In the second configuration the LEDs were allocated along the central row and a parasagittal row in the ipsilateral space. Even though no reaches were performed by the animals, we checked that the two nearest positions in each row were within reaching distance (<30 cm). A total of 101 neurons were tested in both central and contralateral space; 83 of these neurons were also tested in the ipsilateral space.

We studied the neural activity in a time epoch around the saccadic eye movement (perisaccadic activity) and during the subsequent fixation period (fixation activity). A one way ANOVA test performed independently in each row showed, on average, a significant effect (P<0.05) of the depth of fixation in perisaccadic and fixation epochs in about 30% and 40% of V6A cells, respectively. About 15% of the cells were modulated by the depth of fixation in both epochs (Table 1).

**Table 1:** Incidence of V6A neurons modulated by fixation depth in different parts of space and for different time epochs.

Epoch	Central Space	Contralateral Space	Ipsilateral Space
Peri-saccadic only	15 (28/184)	13(23/184)	13 (10/83)
Fixation only	21(39/184)	33(61/184)	27 (22/83)
Perisaccadic and Fixation	16(29/184)	16 (30/184)	14 (12/83)

Percentages and number of neurons (in brackets) with a significant effect (ANOVA, P<0.05) of fixation depth.

### Perisaccadic and fixation activity

**Figure 2:**
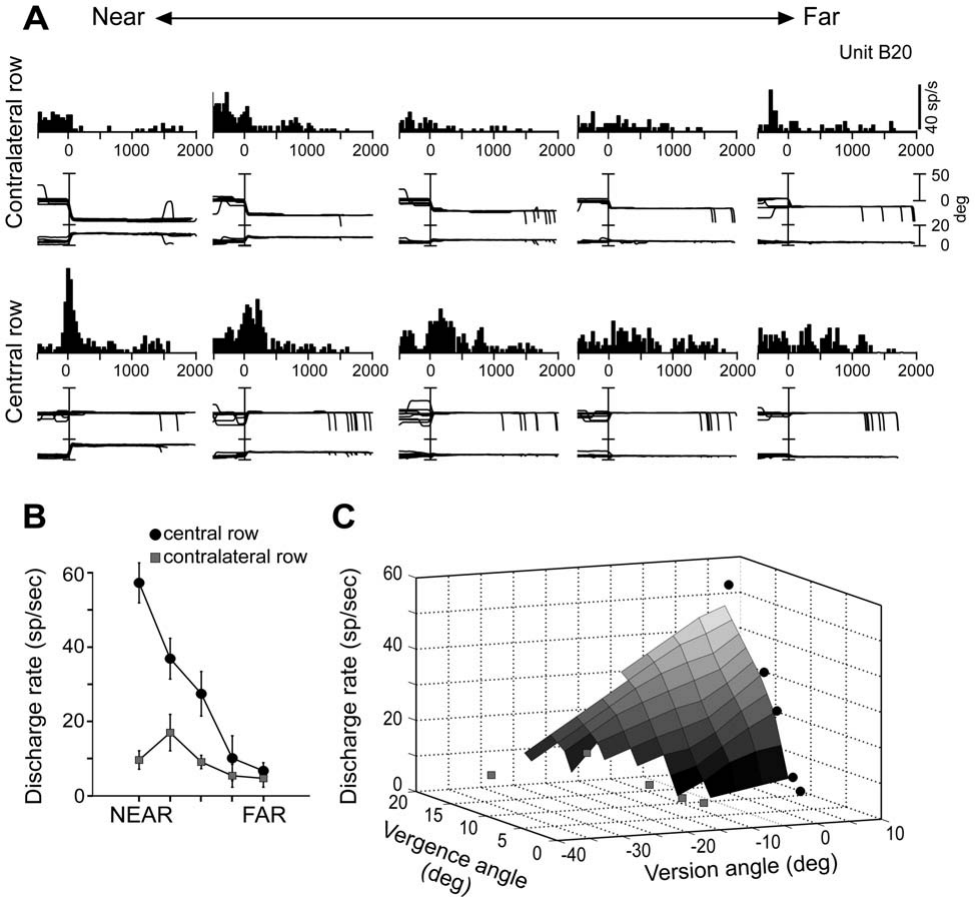
Example of a neuron with perisaccadic activity modulated by depth. (A): Spike histograms of neural responses, eye traces (version upper trace, vergence lower trace) to the five LEDs of the contralateral/central row arranged from near (left) to far (right), aligned at the start of the saccade. This cell shows a clear preference for saccades at the central and near space. (B): Mean± standard error (s.e.m.) discharge rates of the cell in (A) are shown for each LED of the contralateral (white rectangles) and central (black circles) row. (C): Three dimensonal plot obtained by interpolating mean discharge rates for the average value of vergence and version of each LED of the contralateral (white rectangles) and central (black circles) row. Vergence modulates this cell only along the midsagittal row.

Figure 2A shows a neuron that responded with a burst of activity for saccades to LED targets located along the mid-sagittal plane. The perisaccadic responses differed significantly among the targets (ANOVA, P<0.05), the maximum activity being observed when the monkey made saccades to the nearest LED. For this position, the saccade-related response started approximately ∼50 ms before the eye moved and peaked at saccade onset. For the remaining targets of the central line, the peak of the perisaccadic response was slightly delayed and the discharge intensity decreased progressively towards far space. It could be argued that the cell was actually sensitive to the amplitude of saccade instead of the depth, since in order to fixate the nearest LEDs the animal typically made larger saccades than when gazing at the farthest LEDs (starting fixation points were usually far from the animal; see Fig. 2A). However, when the animal shifted its gaze to the LEDs located in the contralateral space the perisaccadic activity was very weak or completely absent, even though the animal made large saccades. Therefore, it was the 3D vector of the gaze shift (towards near locations along the midsagittal plane) that excited the cell and not its amplitude (see also population data on the mean saccadic amplitude in next section).

The tuning curves of the perisaccadic activity of this neuron are reported in Figure 2B, C. The average activity in the perisaccadic epoch increased monotonically from far to near LEDs in the central space (Fig. 2B, black circles), but not in the contralateral space (Fig. 2B, white squares). Figure 2C provides a 3D view of the tuning for fixation depth and direction. This neuron was modulated by depth when gaze was directed centrally, whereas it was insensitive to vergence when gaze shifted contralaterally.

**Figure 3:**
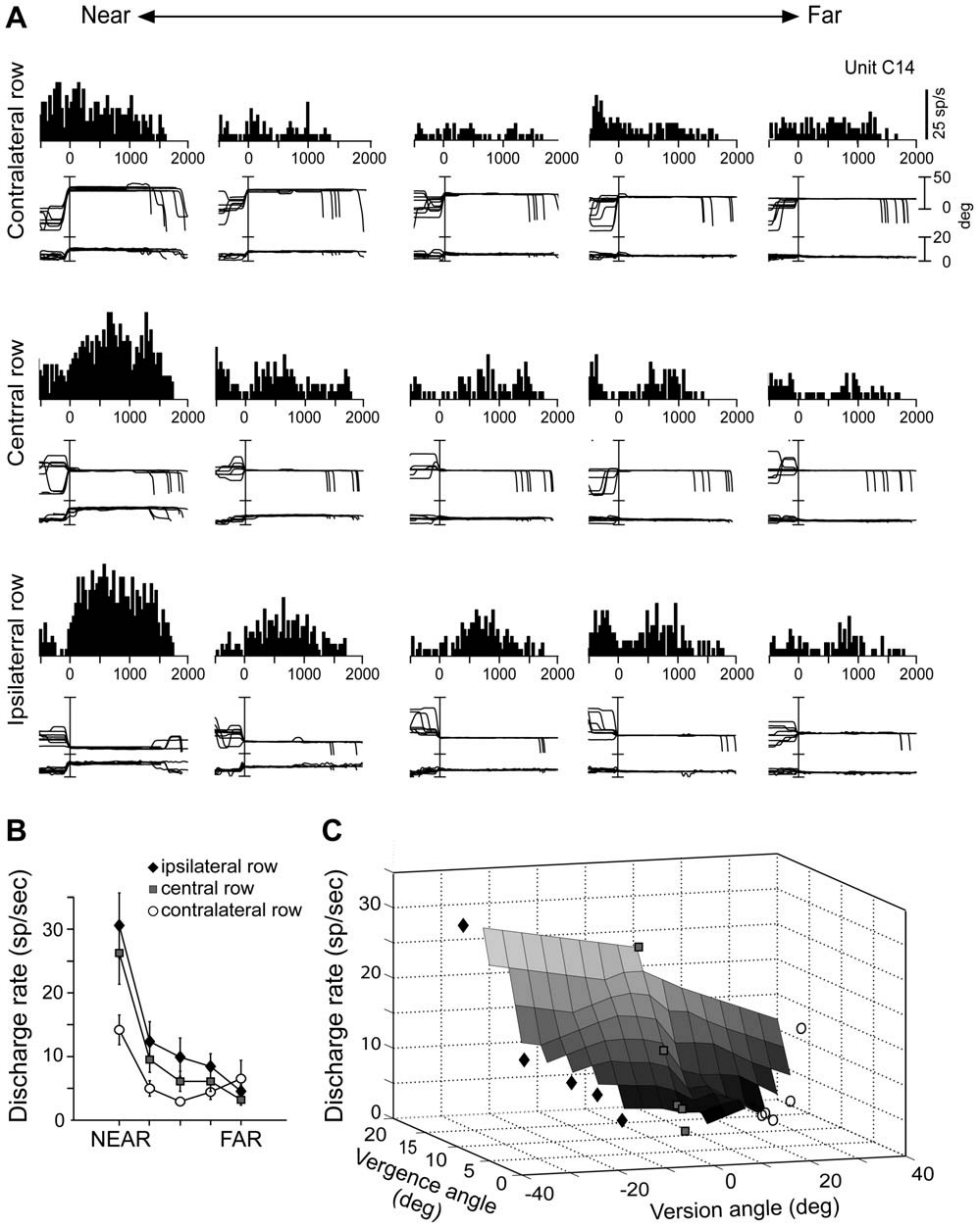
Example of a neuron with fixation activity modulated by depth. (A): Top/Middle/Bottom: neural responses and eye traces to the five LEDs of the contralateral/central/ipsilateral space arranged from near (left) to far (right), aligned at the end of the saccade. This cell prefers targets located at near and ipsilateral space. (B): Mean± s.e.m. discharge rates are shown for each LED of the contralateral (white circles), central (grey squares) and ipsilateral (black diamonds) row. (C): Three dimensional plot obtained by interpolating mean discharge rates for the average value of vergence and version of each LED of the contralateral (white circles), central (grey squares) and ipsilateral (black diamonds) row. Cell discharge reflects a strong tuning by vergence that is influenced by version, so that the cell is activated maximally for fixations on the nearest targets, especially in the ipsilateral space.

Figure 3 shows an example of a neuron that was recorded while the animal performed the task in contralateral, central and ipsilateral space. This neuron was tuned in depth during the fixation epoch, without any evident saccade-related activity. The top part of Figure 3A illustrates the responses of the neuron while the monkey fixated the five LEDs located contralateral to the recording site. The neuron responded strongly when the gaze was directed towards the nearest target, but much less when it was directed towards the remaining positions. The middle part of Figure 3A shows the activity when the monkey fixated the central LEDs. Again, the discharge was stronger for the nearest position and declined gradually when the gazed targets were located farther away. The bottom part of Figure 3A illustrates that fixations in the ipsilateral space evoked higher levels of activity. Yet, again, a trend of decreasing activity was observed from near to far space.

Figure 3B, C shows the tuning of the neuron's mean discharge rate in the 3D space. The mean activity during fixation was higher for the nearest targets in all parts of space tested (Fig. 3B). The neuron was strongly modulated by fixation depth, and this effect was scaled by the direction of gaze (Fig. 3C). Altogether the above data demonstrate that the neuron's fixation activity encodes gaze in 3D space.

The examples reported in Figures 2 and 3 illustrated that the activity of V6A neurons, apart from fixation distance, is also influenced by the gaze laterality, i.e. by the part of space the gaze is directed to. To measure and compare the magnitude of these two effects, a two way ANOVA test was performed on the mean discharge rate of cells that were recorded in both target configurations, that is cells where the whole workspace was explored (N = 83). The results of this analysis are summarized in Table 2.

**Table 2:** Comparison of the effects of fixation depth and gaze laterality on neural activity.

Variables	Epochs	
	Perisaccadic	Fixation
Fixation Depth only	16 (13/83)	14 (12/83)
Gaze Laterality only	36 (29/83)	30 (25/83)
Both	24 (20/83)	45 (37/83)
Interaction only	2 (2/83)	2 (2/83)

Percentages and number of neurons (in brackets) significantly (ANOVA, P<0.05) influenced by each experimental variable. Gaze laterality is defined as gaze direction in space with respect to the recording hemisphere. Gaze directions at monkey's straight ahead are defined as central.

We found that 77% of cells had a statistically significant amount of modulation (P<0.05) in the perisaccadic epoch with respect to at least one of the experimental variables, or their interaction. During fixation, the mean firing rate was significantly modulated (P<0.05) in all but 7 cells tested (91%).

The modulatory effect of direction of gaze (gaze laterality) was more frequent than that of fixation depth, in line with data from parietal area LIP [11]. We also checked whether there was a preferred part of space for eye movements and fixations. A statistical comparison (Bonferroni *post hoc* test) was performed between central, contralateral and ipsilateral gaze positions in the neurons that were significantly modulated by gaze laterality in ANOVA. We found that the directional gaze preference was equally distributed among contralateral, central, and ipsilateral space in both experimental epochs (perisaccadic: 30 contralateral, 33 central, 37 ipsilateral; fixation: 38 contralateral, 29 central, 33 ipsilateral; *χ* ^2^ test between incidence of preference, P>0.05). This result well agrees with previous studies in both macaque V6A and putative human homologues of it [22], [23], [31].

Overall, the two way ANOVA analysis demonstrated that the average spiking rate during perisaccadic and fixation epochs was sensitive to both direction and depth of gaze, with the two types of modulation being quite often present in the same neuron in a simple additive (neurons modulated in both) or multiplicative (neurons with significant interaction) model (Table 2). As a result, V6A activity is modulated by gaze position in 3D space.

### Population responses for fixation depth

**Figure 4:**
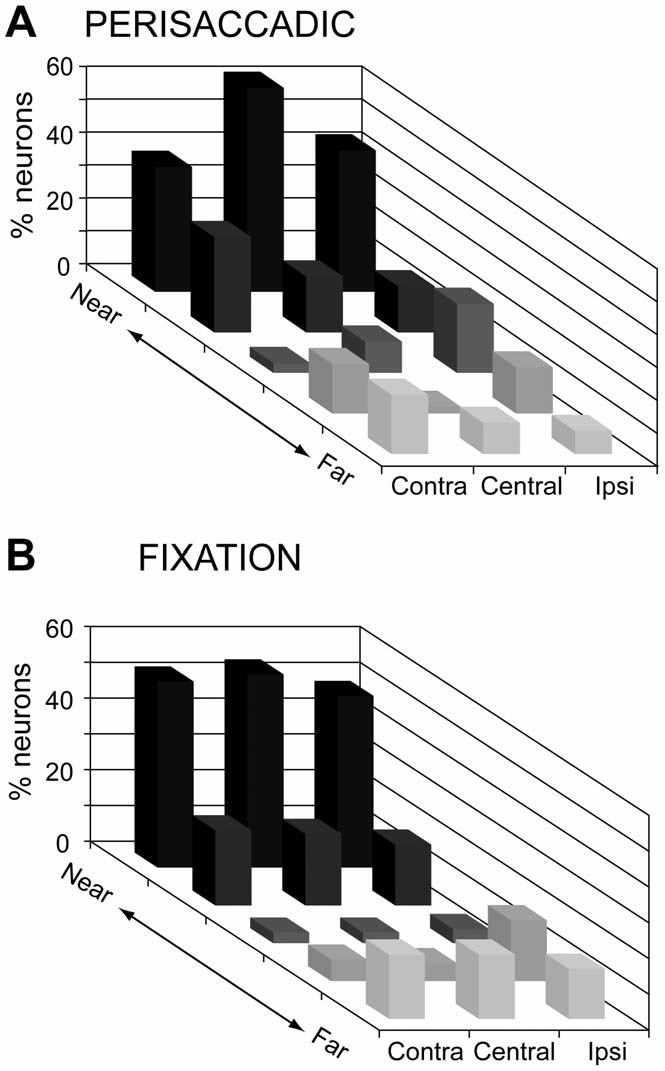
Preferred target positions in depth. Frequency histogram of the positions that neurons preferred in (A) perisaccadic (N = 91) and (B) fixation (N = 167) epochs. Ipsi and Contra indicate fixation position with respect to the recording hemisphere. Center refers to the straight ahead of the monkey. In both epochs there is a clear preference for near, reachable targets across all space.

In order to characterize the modulations with respect to fixation depth, we obtained the distribution of preferred target positions in depth (Fig. 4) by applying a Bonferroni *post hoc* test on the ANOVA significant cells in each row (Table 1). Perisaccadic activity (Fig. 4A) was typically stronger for saccades to the two nearest targets: 60–75% of the cells preferred one of the two nearest LEDs in each row. Considering that only these two LEDs in each row were within reaching distance, we compared the incidence of neurons preferring one of the two nearest (reachable) targets with those that preferred one of the three farthest targets that were outside reachable space. The preference for reachable locations was highly significant (*χ* ^2^ test, two nearest LEDs against the three farthest ones, P<0.0001).

Like neurons showing perisaccadic activity, also the vast majority of neurons modulated during fixation preferred the near, reachable space (Fig. 4B). The preference for fixating targets placed within reachable space with respect to those positioned outside it was again highly significant (*χ* ^2^ test, two nearest LEDs against the three farthest ones, P<0.0001). Thus, at population level, perisaccadic and fixation activity of V6A neurons are aligned in representing mainly the 3D space within reaching distance.

**Figure 5:**
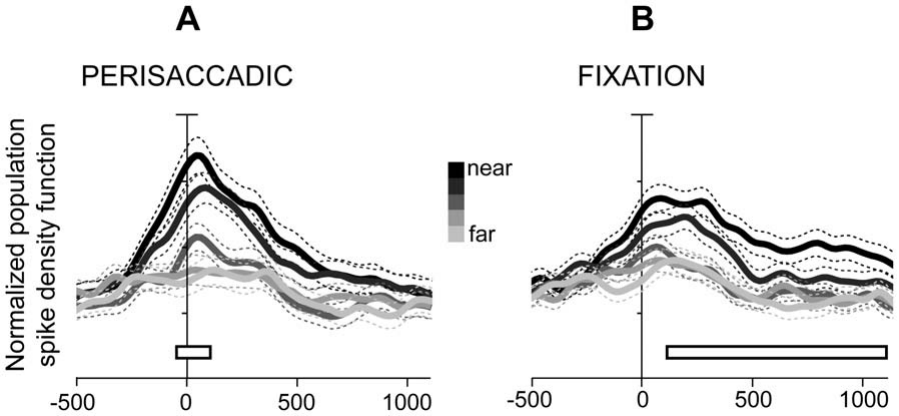
Preference for near space at the population level. Population activity per each LED position (different tones of grey) of V6A cells modulated in (A) perisaccadic (N = 132) and (B) fixation (N = 193) averaging across all rows. Activity is expressed as averaged normalized SDF (thick lines) with variability bands (s.e.m., dashed lines) and is aligned at saccade onset in both (A) and (B). Each modulated cell was taken into account five times, once for every LED position. The peak of the SDF curve of the LED with maximum activity was set to 1 (or 100%) and was used to normalize the activity curves of the other targets. Accordingly, the population activity of each target is expressed as percentage of the averaged normalized activity. White rectangular boxes indicate the time intervals used at the permutation test (two nearest targets always different form the farthest ones, P<0.05, see text); vertical axis: 10% of normalized activity per division and axis origin corresponds to 20% of normalized activity.

The preference for near space was also evident when we calculated the average normalized spike density function (SDF) of each position in depth (Fig. 5). For each modulated cell, the SDFs of different LED positions were grouped together and averaged according to their distance from the animal (i.e. nearest, second near etc.). In Figure 5, every SDF curve represents the average activity of each target across contralateral, central and ipsilateral space. The cumulative SDFs of cells modulated in perisaccadic epoch (Fig. 5A) showed that the average responses were stronger for the nearest two targets (darkest lines) and weaker for the three farther ones. The curves of the two nearest targets started to significantly diverge from the others well before the saccade onset (on average at -150 ms, pairwise sliding permutation test P<0.05; see Methods), thus suggesting that neurons begin to encode the eye movement to near space before saccade occurrence, at the time of saccade planning. The curves of the two nearest targets were found to be significantly different from those of the three farthest ones also during perisaccadic epoch (pairwise fixed-interval permutation test P<0.05). Likewise, the population response during fixation epoch (Fig. 5B) was significantly higher for the two nearest targets compared to the three farther ones (pairwise fixed-interval permutation test, P<0.05). Interestingly, the near responses differed more notably from the far responses in the first 500 ms of fixation (Fig. 5B). By the time course of the population discharges it is evident that the 2 components (perisaccadic and fixation) are often coupled and that both of them preferentially encode the near space.

In our experiment the initial eye position varied between the trials. As a result, the same target could be fixated with saccades of different amplitudes of version and vergence. It could be argued that an amplitude effect could be responsible for the neural modulations found in V6A cells. To check this we performed a population analysis on the mean saccadic amplitude.

Regarding the version amplitude, saccades to central targets were on average shorter than those to the lateral ones (mean +/− S.D. for central, contralateral and ipsilateral targets were 8+/−7.7 deg, 18.8+/−11.7 deg, 17.8+/−12.6 deg; ANOVA P<0.05, Bonferroni post-hoc central-contralateral, central-ipsilateral, P<0.05). This resulted from the fact that the animals tended to gaze at straight ahead positions before the targets appeared. Similarly, the initial depth of fixation was more frequently closer to the far space so the fixation of nearer targets was associated with larger vergence saccades (mean +/− S.D. for near, 2^nd^, 3^rd^, 4^th^, and far targets grouped across space were 7.8+/−2.9 deg, 4+/−1.8 deg, 1.8+/−1.6 deg, 0.5+/−1.6 deg, −0.1+/−1.5 deg ; ANOVA P<0.05, Bonferroni post-hoc all group comparisons, P<0.05). To see whether the changes in vergence and/or in version accounted for the neural discharges, we used multilinear regression to determine their effect on perisaccadic and fixation activity. This analysis was applied on the subset of neurons that were tested in the whole workspace and found to be modulated in ANOVA (P<0.05, Table 2). In Table 3 the mean values of the normalized linear coefficients and the numbers of cells fit for each variable and epoch of activity are listed. In both perisaccadic and fixation epochs, the vectors of vergence (delta vergence) and version (delta version) change had a moderate linear effect (0.33–0.35) on activity in about the two thirds of modulated cells. A linear correlation of activity with the amplitude of the version change (delta version amplitude) was found in a minority of modulated cells. Importantly, the delta vergence coefficients could equally be positive or negative (perisaccadic epoch: 13 positive, 9 negative; fixation epoch: 16 positive, 16 negative), thus showing a similar incidence of neurons that preferred convergence and divergence saccades (*χ* ^2^ test, P>0.05). Likewise, the coefficients of delta version vector and amplitude were evenly distributed (perisaccadic epoch: 9 rightward, 14 leftward, 9 large, 10 small saccades; fixation epoch: 16 rightward, 25 leftward, 13 large, 6 small saccades, *χ* ^2^ test, P>0.05).

**Table 3:** The effect of saccadic amplitude on neural activity.

Variables	Epochs	
	Perisaccadic	Fixation
delta Vergence	0.33 (22/33)	0.35 (32/49)
delta Version	0.39 (23/49)	0.35 (41/62)
delta Version amplitude	0.29 (19/49)	0.28 (19/62)

Mean absolute standardized coefficients of the linear regression with the number of neurons (in brackets) significantly fit for each saccade amplitude parameter and analysis epoch. Delta vergence: changes in vergence from initial fixation to target fixation; delta version: changes in version; delta version amplitude: absolute changes in version.

The multilinear regression analysis showed that the modulation of neural activity can be only partially explained by the version and/or vergence changes, and the amplitude of version change was significantly correlated with the neural discharges only in a minority of cells. Furthermore, only in half of the cases activity increased for convergence/larger saccades. This suggests that he preferential encoding of near targets is a phenomenon not entirely attributable to saccadic amplitude.

**Figure 6:**
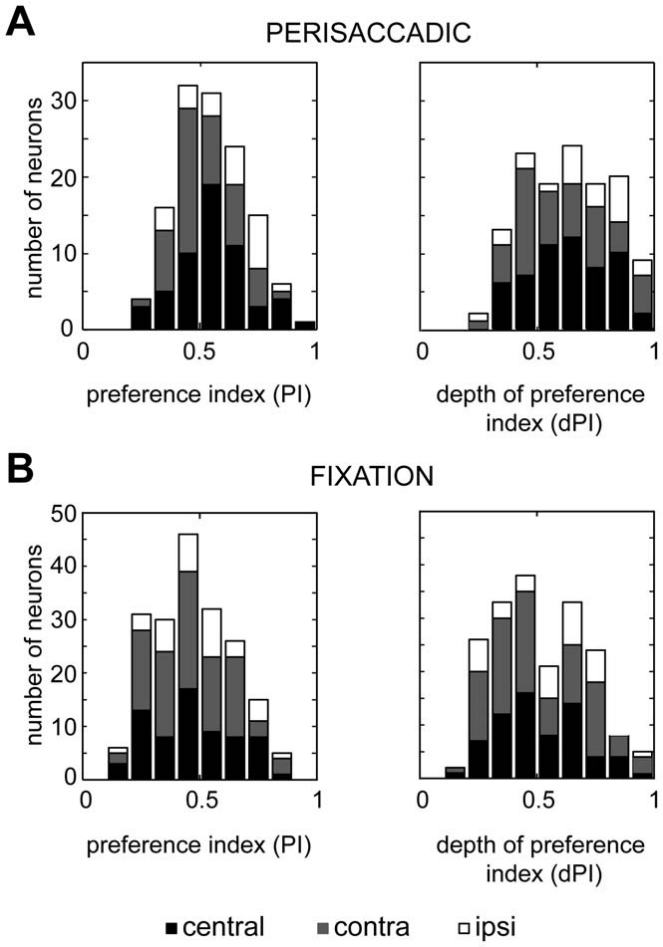
Selectivity and strength of modulation for depth in V6A. Frequency distributions of preference index (PI), and depth of preference index (dPI) of the cells modulated in perisaccadic (A) and fixation (B) epochs in central, contralateral and ipsilateral space.

To investigate the selectivity and strength of neural modulations in 3D space, we computed two indexes: the preference index (PI), and the depth of PI (dPI), as detailed in Methods. Both indexes can range between 0 and 1. A PI value of 1 indicates that a neuron prefers only one fixation condition, whereas a value of 0 indicates equal response for all conditions. A dPI value of 1 indicates a maximum modulation of responses between the preferred and non preferred condition, whereas a value of 0 indicates no modulation. The PI index of cells with activity significantly modulated in perisaccadic (Fig. 6A, left) and fixation (Fig. 6B, left) epochs in all parts of space ranged from 0.16 to 0.92 (mean ± S.D., 0.50±0.16). Comparison of their distributions for central space revealed a significant difference between the perisaccadic and fixation epoch (Kolmogorov-Smirnov, two-tailed, P<0.01). This difference became more pronounced when, besides the central space, the distributions of the contralateral and ipsilateral space were also taken into account (Kolmogorov-Smirnov, two-tailed P<0.0001).

The right column of Figure 6 shows that the dPI index of cells modulated in all space varied between 0.12 and 0.98 (mean ± S.D., 0.56±0.19). The index values of perisaccadic responses for central targets (Fig. 6A, right) were significantly different from those of fixation (Fig. 6B, right; Kolmogorov-Smirnov, two-tailed P = 0.001). When the dPIs of cells modulated in contralateral and ipsilateral space were also included, this difference became highly statistically significant (Kolmogorov-Smirnov; two-tailed P = 0.0005).

The above indexes were compared pairwise in individual neurons showing both significantly modulated perisaccadic and fixation responses (N = 76 pairwise comparisons in all rows). Again, the perisaccadic responses had significantly higher PI and dPI values (Wilcoxon rank sum, two-tailed P<0.001 for PI, P<0.0001 for dPI, including responses in all rows). In conclusion, the analysis of modulation indexes revealed that both the selectivity of fixation depth effect and the strength of neural response modulation were more evident around saccade occurrence than during fixation.

### Near and Far cells

**Figure 7:**
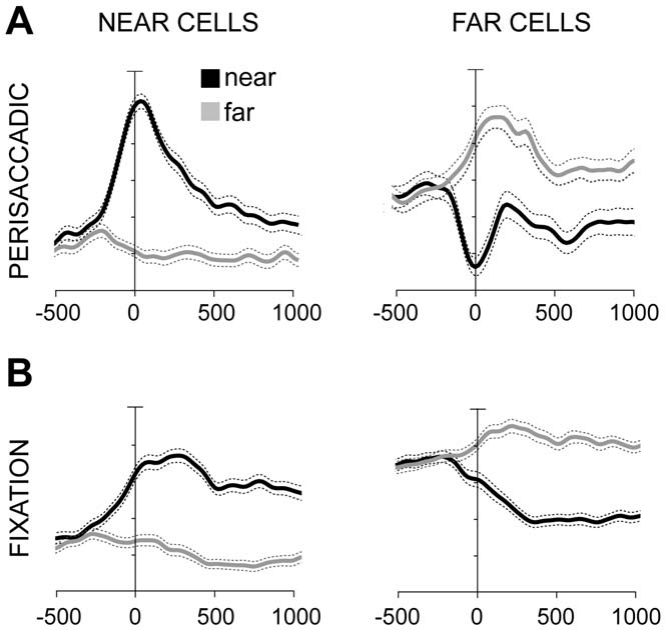
Population activity of “Near” and “Far” cells. (A). Average activity represented as averaged normalized SDF (ordinate) for near (black) and far targets (grey) in V6A cells with preference in perisaccadic epoch for the two nearest targets, “Near”cells (N = 132) and for the three farthest targets, “Far” cells (N = 51). (B). Average activity of Near cells (N = 230) and Far cells (N = 89) in fixation epoch. In both A and B activity is aligned at saccade onset. Other details as in Figure 5.

Cells sensitive to the depth of fixation were classified as “Near” or “Far” cells according to their preference during the perisaccadic and fixation epoch (Fig. 4). We defined as “Near” the neurons maximally activated by one of the two targets, in each row, nearest to the animal. “Far” were the neurons that preferred one of the three targets in each row located in the animal's extrapersonal space (unreachable space). Figure 7 shows the population SDFs of Near and Far cells in perisaccadic (Fig. 7A) and fixation (Fig. 7B) epoch. The black lines indicate the average activity when the animal fixated the nearest targets; the gray lines the activity when it fixated the farthest targets. Near neurons (Fig. 7, left), started to change their activity according to their preference about 150 ms before the saccade onset (pairwise sliding permutation test, P<0.05), whereas Far cells (Fig. 7, right) about 100 ms before the saccade (pairwise sliding permutation test, P<0.05). Interestingly, Far cells showed a robust, phasic inhibition in coincidence with the saccade to near targets (Fig. 7A, right, black line). Their fixation activity dropped to a minimum about 300 ms after near targets were gazed, and was tonically suppressed for the rest of fixation (Fig. 7B, right, black line). The above results revealed the presence of a different coding mechanism in the Near and Far cells. Regarding the perisaccadic responses, Near neurons showed strong excitation when the animal gazed targets within reaching distance and slight inhibition when the eyes were directed towards unreachable targets. Far cells, conversely, increased their activity for gazing targets located in the extrapersonal space and were strongly suppressed for gazing near, reachable locations. Considering the fixation responses, Near cells showed an increase of activity for near space and remained unmodulated for far space. On the other hand, in Far cells the activity remained quite constant for far space and was strongly inhibited for near space. In other words, it seems that the activity is not modulated during far fixations in both types of cells, whereas it is strongly excited and strongly inhibited for near space in Near and Far cells, respectively.

### Anatomical location of recorded cells

**Figure 8:**
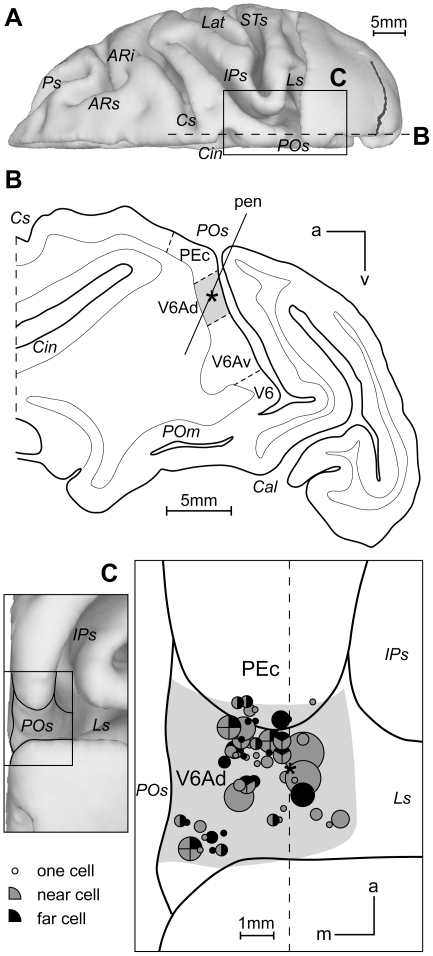
Recording sites and preference for near and far space. (A) The location of area V6A in the parieto-occipital sulcus (POs) is shown in a dorsal view of a hemisphere reconstructed in 3D using Caret software (http://brainmap.wustl.edu/caret/). (B) Representative parasagittal section at the level of the dashed line of panels A and C showing the dorsoventral extent of recorded region. Grey area: extent of V6Ad in that section. Asterisk: recording site of two near cells. (C) Enlarged view of the recorded region. Each circle represents the projection on the dorsal surface of a single penetration. Circle diameter is proportional to the incidence of preference for near (black) and far (gray) space. Asterisk: as in B. Dashed line: level of the section shown in B. Ps, principal sulcus; ARi, arcuate sulcus inferior ramus; ARs, arcuate sulcus superior ramus; Cs, central sulcus; Cin, cinguate sulcus; Lat, lateral sulcus; STs, superior temporal sulcus; IPs, intraparietal sulcus; Ls, lunate sulcus; POm, medial parieto-occipital sulcus; Cal, calcarine sulcus; m:medial; a:anterior; d:dorsal.

The anatomical reconstruction of the recorded sites was performed using the cytoarchitectonic criteria described by Luppino et al. [32], and the functional criteria detailed in [21], [33] , and summarized in the Methods. Figure 8A shows the location of the parieto-occipital sulcus (POs), where area V6A is located, in a 3D reconstruction of one hemisphere. Figure 8B shows a parasagittal section cut halfway the medio-lateral extent of POs (see dashed lines in Fig. 8A and C), and the extent of the dorsal sector of V6A (V6Ad, grey shaded area) where all the neurons of this study were recorded. In this penetration, two neurons preferring near space were recorded, whose location is indicated by an asterisk in Figure 8B and C. In other cases, near and far cells were encountered along the same penetration without any systematic relationship with the depth of the electrode tip. In the left part of Figure 8C, a dorsal and posterior 3D view of the reconstructed POs is illustrated. In the right part of Figure 8C, the region of POs is shown at higher magnification. Grey shaded area shows the mean borders of V6Ad in the two monkeys. Circles of different size indicate the relative incidence of modulated neurons. Although near cells (preferring peripersonal space) were more represented than far cells, the two cell populations were not anatomically segregated. In other words, there was not any topographic organization in V6Ad: neurons preferring near and far space were intermingled.

## Discussion

The present study is the first to provide evidence that neurons of the medial PPC are sensitive to fixations and eye movements in depth, and are more activated by gaze positions in the near, peripersonal space. In fact, the large majority of V6A neurons (60–75%) preferred the near, peripersonal space, a minority (15–35%) the far, extrapersonal space, and occasional units intermediate distances. Here we discuss the possible functional implications of this representational bias.

### Encoding of fixation depth in the cortex

The effect of fixation depth on neural activity of monkey PPC has been reported in some previous studies [10], [12], [13]. Sakata and colleagues [10], tested the influence of fixation depth on neuronal activity in area 7a. The range of fixation distances they explored was larger than that we have explored in the present experiments (10–160 cm *versus* 15–75 cm in our configuration). These authors reported that in area 7a the majority of visual fixation neurons preferred distances less than 50 cm. They also observed several units with preferred distances greater than 100 cm, and only a few units firing maximally at intermediate distances.

Similarly, our study revealed the existence of two subpopulations of V6A neurons modulated by depth. “Near” cells, representing the majority of modulated neurons, increased their firing rate for saccades and/or fixations of targets located in the peripersonal space. “Far” cells, representing a smaller proportion, exhibited excitation during eye movements and fixations in the extrapersonal space, as well as inhibition for targets located in the peripersonal space. Our study further specified that the preferred fixation distances mainly extend within the limits of reachable space (up to 30 cm from the body). The fixation distance of 45 cm, which is just outside the monkey's reachable space, was the least represented in our cell population. This ‘gap zone’ of fixation representation, between the reachable and unreachable space, could enhance the signalling of target presence either within or beyond animal's reachable space.

Genovesio and Ferraina [12] reported that in about one third of LIP neurons the vergence had a significant effect on the planning activity for conjugate eye movements, with about 70% of LIP neurons preferring saccades in near space. However, as they explored only the peripersonal space (range of vergence angles: 6°–13°), we do not know whether also in area LIP there is a preference for reachable *versus* non-reachable space. In addition, due to differences in the task (conjugate vs disconjugate saccades) and in activity epoch (planning vs peri- or postsaccadic), their study is qualitatively different from ours and does not allow for further comparisons. Bhattacharyya and co-workers [13] have recently reported that vergence modulated the planning activity of a large number (74%) of arm reaching neurons in area PRR. However they did not address the question whether or not there was a preference for near vergence.

In general, the present and above studies suggest a modulatory effect of fixation distance on neuronal activity is a common feature among anatomically interconnected parietal areas [34] that represent nodes of the dorsal visual stream [28]. Interestingly, however, the influence of a vergence signal on fixation activity has been also reported in the primary visual cortex [16] and in area V4 [17], i.e. in the ventral visual stream, and in both cases the majority of neurons preferred the near space.

A strong result of our study was that V6A neurons were mainly activated by near fixations. We tested the effect of fixation depth in the lower part of the working space, at shoulder level. This region of space is where most of the primate visually guided motor behavior takes place [18] and where 3D gaze shifts most frequently occur [35]. The hands are usually located in the lower visual field, and, in turn, the lower visual field is particularly well-represented in areas of the dorsal visual stream, including V6A [21], [36]. There is also a growing body of evidence suggesting a functional coupling of near and lower space. Convergence eye movements were found to be better synchronized with downward saccades than with upward ones [37], which agrees with the fact that we usually look down to fixate near targets. In line with this assumption, transcranial magnetic stimulation of human PPC significantly increased the disconjugacy during downward saccades [38]. The near/lower and far/upper coupling in spatial arrangement of targets has been employed in several recent human psychophysical and functional neuroimaging studies. Some of them suggested the involvement of putative human homologues of macaque V6A in depth processing and in encoding of peripersonal space [4], [5], [30].

We are aware that in our experimental configuration the nearer targets were associated with more downward eye positions. Therefore, the higher activity observed for nearer targets could be due, at least in part, to the difference in elevation angle. Nevertheless, in previous work that tested the eye position sensitivity of V6A neurons no significant bias for downward eye positions was observed [22], [23]. Therefore, we believe that the higher activity observed for nearer targets is due to the high vergence angles used to fixate them. Studies more specifically directed to this issue are needed to prove this hypothesis. Another account of our major finding could be given by the fact that the fixation of nearer targets was on average associated with larger saccade amplitudes, both in vergence, and in version for the lateral space. Although we cannot completely exclude that possibility, our regression analysis showed a roughly equal proportion of neurons activated by convergence and divergence and the same was also true for the neurons preferring large and small version amplitudes. In addition, linear correlations of activity with saccadic amplitude were observed in a majority, but not in all the modulated neurons tested.

The depth preference of V6A cells found in fixation activity, with the targets being already foveated, could not be accounted for by disparity stimulation, unless we consider that all cells were tuned for zero disparity. Even in that case, the different response for fixating near and far targets would mean that vergence acts as gain field mechanism on the disparity tuning. For the perisaccadic activity we cannot exclude a contribution of disparity stimulation. However, like in our example neuron of Figure 2, for most of our neurons the responses were better aligned on the saccadic event than on the switching on of the target. In addition, a considerable fraction of neurons modulated in perisaccadic epoch were also modulated during fixation (Table 1), making it quite unlikely that they responded to two different values of disparity. Another possible explanation of our results is that they could reflect the overrepresentation of the lower visual field in V6A [21]. If that was the case, in the perisaccadic epoch we should have found a prevalence of neurons preferring the contralateral space, where the majority of the visual receptive fields of V6A neurons are located [21]. In contrast, the statistical comparison of preferred gaze positions did not show any laterality effect. Furthermore, area V6A is known to contain non visual neurons influenced by gaze position in 2D space in complete darkness [22], and many V6A neurons had strong background discharges in the dark following spontaneous eye movements (present results; see example neurons in Figs. 2 and 3).

In natural conditions, when we want to grasp an object, the eyes fixate on the target first and then the hand starts to move [39], [40], [41]. Fixation of the target requires a saccadic eye movement performed in the right direction and depth that “captures” the target and brings it onto the fovea. V6A perisaccadic activity might encode the saccadic event and it could be used to modulate the activity of arm reaching neurons highly represented in V6A [42]. It is worthwhile to note that information about eye position during the period around saccade is critical for the motor center that controls the hand, because the retinal coordinates of the target change with the saccade. In this context, early fixation activity could constitute a fixation-for-reaching signal that brings the new retinal coordinates of the target to the arm reaching neurons

### Encoding of depth and peripersonal space in human medial PPC

Damages to the medial PPC in humans have been specifically associated with depth deficits affecting visually guided reaching and pointing towards targets arranged in a configuration similar to that the one used in the present work [4], [5]. Baylis et al. [4] reported a case study of a patient with bilateral medial PPC lesion that exhibited significantly more errors in depth than in direction during reaches to targets placed on a tilted panel. The above findings were confirmed and extended in a more recent study of an optic ataxic patient tested on pointing movements to targets located on a table [5]. These authors reported that pointing movements were more impaired in the sagittal than in the frontoparallel plane, with pointing towards the body being the most affected one.

In a functional imaging study in humans, Quinlan and Culham [43] described a region in the human dorsal parieto-occipital cortex that was more strongly activated when subjects viewed moving or stationary stimuli located in near space than in intermediate or far space. The same result was obtained when subjects fixated small LEDs placed at the same range of distances (near and far) and it was attributed the to the oculomotor near response, most probably to the convergence of the eyes [43], [44]. These fMRI data are in line with the present findings, thus indicating a functional homology of human and monkey medial PPC in encoding the near space.

A subsequent work [30] reported that passive viewing of graspable objects placed within the reachable space evoked higher activity in the superior part of parieto-occipital cortex (SPOC) compared to viewing the same objects placed in non reachable space. It is worthwhile to note that Gallivan et al. [30] used an experimental setup very similar to that we used in the present work, with near objects placed downwards compared to the far objects. Fixation was held constant at a medium/far location, so the stronger activation of SPOC by objects located near was not ascribed to the convergence of the eyes but to the reachability of targets, which could be extracted by the combination of gaze and target depth signals. The SPOC region, which was also activated during arm reaching movements [45], includes the cortex anterior to the human homologue of V6 [45], [46], i.e. the putative area V6A.

The representation of peripersonal space in several brain areas of humans and primates is considered to be formed through the convergence of visual and somatosensory information [47], [48], [49] Area V6A has been proved the earliest node of the dorsal visual stream where visual, eye and arm position related information converge [24], [33], [50] . Therefore, the multisensory encoding of space is likely to be realized in this cortical region. The strong preference for reachable space during the perisaccadic and early fixation periods could be exploited by V6A neurons to form neural representation of space where to reach or interact with objects. A complementary -not alternative- hypothesis is that this preference might reflect an attentional shift to the location of salient targets that can be reached out or grasped. In this context, the near response would function as an anticipatory feature for object-oriented actions.

The present study suggests a novel role for the medial posterior parietal area V6A in constructing a 3D representation of the visual world. It has been shown that the two dimensional location of visual targets [22], some of them in spatiotopic coordinates [51] is encoded in V6A. Here we show that many V6A neurons also encode the spatial locations that the animal is going to gaze, or that it is actually fixating in 3D space, with a prevalence of cells preferring reachable locations. Since V6A has been also found to contain many reach-to-grasp neurons [28], the data of the present study give strong support to the view that V6A plays a key part in the sensory-to-motor transformations that control reach-to-grasp arm movements.

## Materials and Methods

### Ethics Statement

Two male *Macaca fascicularis* monkeys were studied. Experiments were approved by the Bioethical Committee of the University of Bologna and authorised by the Ministry of Health (Permit N^o^ DM 47/2008-B, 6/4/2008). Experiments were performed in accordance with national laws on care and use of laboratory animals, with the European Community Council Directive of 24th November 1986(86/609/EEC), and with the recommendations of the Wheatherall report (‘TheUse of non-human primates in research’). All procedures used have been approved and supervised by the Central Veterinary Service of the University of Bologna.

### General procedures

In each monkey, a head restraint system and a recording cylinder were surgically implanted in asepsis under general anesthesia (sodium thiopenthal, 8 mg/kg/h, *i.v*.) following the procedures reported in Galletti et al. [22]. Adequate measures were taken to minimize pain or discomfort. A full program of postoperative analgesia (ketorolac trometazyn, 1 mg/kg *i.m.* immediately after surgery, and 1.6 mg/kg *i.m.* on the following days) and antibiotic care [(Ritardomicina ® (benzatinic benzylpenicillin plus dihydrostreptomycin plus streptomycin)] 1–1.5 ml/10 kg every 5–6 days) followed the surgery.

Single cell activity was recorded extracellularly from area V6A [20], [21]. We performed single microelectrode penetrations using home-made glass-coated metal microelectrodes with a tip impedance of 0.8–2 MOhms at 1 KHz, and multiple electrode penetrations using a 5 channel multielectrode recording system (Mini Matrix, Thomas Recording, GMbH, Giessen, Germany). The latter electrodes were quartz-platinum/tungsten fibers with an impedance of 0.5–2 MOhm at 1 kHz (Thomas Recording). Electrode signals were amplified (gain 10,000) and filtered (bandpass between 0.5 and 5 kHz). Action potentials in each channel were isolated with a dual time-amplitude window discriminator (DDIS-1, Bak electronics, Mount Airy, MD, USA) or with a waveform discriminator (Multi Spike Detector, Alpha Omega Engineering, Nazareth, Israel). Spikes were sampled at 100 KHz. Signals from both eyes were recorded simultaneously with an infrared oculometer (ISCAN Inc., Woburn, MA, USA) at a sampling rate of 100 Hz.

Location of area V6A was initially identified on functional grounds during recordings [21], [24], [33]. To ascribe a recording site as being potentially located in V6A the following tests were performed: a) visual responsiveness, and b) somatosensory and skeletomotor responsiveness. The visual responsiveness of neurons was first tested with simple visual stimuli as light/dark borders, light or dark spots and bars of different size, orientation, direction and speed of movement. Cells with no responses were further studied with more complex stimuli such as light/dark corners of different orientation, direction and speed of movement, shadows of irregular contours (e.g. waving hand shadows) changing in size and shape. The mixture of visual and non visual neurons in a single or in adjacent sites and the presence of visual neurons that could be driven only by complex stimuli were used as defining criteria. In several sites, somatosensory responsiveness was studied with light touching, deep pressure or joint manipulation of several body parts. In addition the monkey performed active arm movements to take pieces of food. The presence of responses to passive or active arm manipulation was used as an additional criterion [24], [33] .

### Histological reconstruction of electrode penetrations

At the end of the electrophysiological recordings, a series of electrolytic lesions (10 µA cathodic pulses for 10 sec) were performed at the limits of the recorded region. Then each animal was anesthetized with ketamine hydrochloride (15 mg/kg, i.m.) followed by an intravenous lethal injection of sodium thiopental. The animals were perfused through the left cardiac ventricle with the following solutions: 0.9% sodium chloride, 3.5–4% paraformaldehyde in 0.1 M phosphate buffer, pH 7.4, and 5% glycerol in the same buffer. The brains were then removed from the skull, photographed, and placed in 10% buffered glycerol for 3 days and in 20% glycerol for 4 days. Finally, they were cut on a freezing microtome at 60 microns in parasagittal plane. In all cases, one section every five was stained with the Nissl method (thionin, 0.1% in 0.1 M acetate buffer, pH 3.7) for the cytoarchitectonic analysis. Procedures to reconstruct microelectrode penetrations and to assign neurons recorded in the anterior bank of the parieto-occipital sulcus to area V6A, were as those previously described by our group [20], [21], [33], [52] . Briefly, electrode tracks and the approximate location of each recording site were reconstructed on histological sections of the brain on the basis of the lesions and several other cues, such as the coordinates of penetrations within recording chamber, the kind of cortical areas passed through before reaching the region of interest, the depths of passage points between gray and white matter. All neurons of the present work were assigned to area V6Ad on the basis of their location in one of the two cytoarchitectonic sectors of V6A following the criteria defined by Luppino and coworkers [32]. This process is presented in more detail in a recent work by our group [33].

### Behavioral task

Each monkey sat in a primate chair with the head restrained and faced a horizontal panel located 10 cm below the level of its eyes, roughly at shoulder level (Fig. 1A). Ten circular light emitting diodes (LEDs, 5 mm diameter), mounted on the panel at different distances from the eyes, were used as fixation targets. The target LEDs were arranged in two rows (5 LEDs in each row): one central, along the sagittal midline and one lateral, 17 cm to one side. Along each row, LEDs were spaced 15 cm apart. The frontoparallel distance of the nearest LED of the central and lateral rows from the eyes was 15 cm (Fig. 1A). During the experiment, the LED panel was first positioned so that the lateral LEDs were in the contralateral space with respect to the recording site. In cases where a cell was held in isolation long enough, after recording its activity in this configuration the panel was translated to a symmetrically ipsilateral position, so to present the LED targets in central and ipsilateral space. The interocular distance of the monkeys M1 and M2 was 35 mm and 32 mm, respectively, so the vergence angle of central LEDs were 14°, 9°, 6°, 5° and 4° for monkey M1 (Fig. 1C), and 12°, 7°, 5°, 4° and 3°, for monkey M2. The corresponding vergence values for the LEDs of the lateral rows were 11°, 8°, 6°, 5° and 3.5° for M1, and 10°, 6°, 4°, 3° and 2.5° for M2. The horizontal version of the lateral LEDs from the nearest to the farthest one was 36°, 29°, 21°, 17° and 14°. Since the LEDs were located at the same horizontal level below the eyes the elevation angle was a linear function of vergence. In the central row from the nearest to the farthest LED the elevation angle was 33°, 18°, 13°, 9° and 8°, respectively, whereas the corresponding values for the lateral rows were 24°, 16°, 12°, 9° and 7°.

Although the task did not require the monkey to reach and touch the LEDs (see below), we checked that the monkeys were able to reach only the two nearest ones in each row. The remaining LEDs of each row were beyond their reachable space. The reachability check was carried out only once for each monkey, at the very beginning of training.

Monkeys were trained to fixate in darkness one of the LEDs for a variable period of time. The time sequence of the task and an example of a disconjugate eye movement are shown in Figure 1B. A trial began when the monkey pressed a home button (HB) next to its chest (HB press) and had to keep it pressed while performing the task. After a 1000 ms interval, during which the animal was free to look anywhere, one of the LEDs turned on (LED). The animal had up to 600 ms to make an eye movement to fixate the LED, otherwise the trial was automatically aborted. After either 1 or 1.5 sec from the start of fixation, the color of the LED changed from green to red and this signalled the end of the task (Task). The monkey had to release the button in order to receive reward (HB release). The monkey's arm ipsilateral with respect to the recording hemisphere was restrained, so the animal pressed and released the button with the contralateral hand.

Fixation LEDs were chosen in random order trial by trial. While recording a neuron, each LED was presented ten times. Eye movements were a combination of conjugate (version) and disconjugate (vergence) movements. Because the monkey was free to look anywhere at the beginning of the task, the initial position of the eyes varied from trial to trial. During fixation, eye position was monitored by an electronic window (2°×2°) centered on each LED. Stimulus presentation and animal's behavior, including eye position signals, were monitored in real time with the use of custom software written in Labview (National Instruments, Austin, TX, USA), as previously described [53].

Before each recording session, the monkey was required to perform a calibration task on each eye separately. In this task, the monkey fixated in sequence ten LEDs that were mounted on a frontoparallel panel at a distance of 15 cm from the eyes. For each eye, signals to be used for calibration were extracted during fixation of five LEDs, one central aligned with the eye's straight ahead position and four peripheral placed at an angle of +/−15° (distance: 4 cm) both in the horizontal and vertical axes. From the two individual calibrated eye position signals we derived the mean of the two eyes (the conjugate or version signal), and the difference between the two eyes (the disconjugate or vergence signal) using the equations: version = (R+L)/2 and vergence = R-L, where R and L was the position of the right and left eye, respectively.

### Data analysis

Rasters of spiking activity were aligned on specific events of the task sequence. Mean discharge rate was quantified in several epochs: ‘Perisaccadic’, from 50 msec before the onset of the saccade till 50 msec after the end of it; ‘Fixation’, from 50 msec after the end of the saccade till the go signal to release the button (1000 or 1500 ms after saccade offset, randomly selected by the computer).

The onset and offset of a saccade were defined as the time point where the velocity of version movement exceeded or dropped below the 15% of the peak velocity, respectively. Trials with eye movements in the wrong direction, anticipatory movements (latency shorter than 100 ms), slow movements (latencies longer than 600 ms), or movements combined with blinks were rejected.

In order to study the effect of fixation in depth on neural activity while minimizing the well known influence of eye position in the fronto-parallel plane [22], we performed an ANOVA test separately for the central, contralateral, and ipsilateral row (P<0.05). In each row, cells with a significant ANOVA were further tested with a Bonferroni *post hoc* correction to select the neuronal spatial preference. For statistical purposes, cells were included in the ANOVA analysis of a given row when there were at least seven trials for each of the five LEDs of the row. Neurons with mean firing rate lower than 3 spikes/s were excluded from the analysis.

For the significantly modulated neurons in each row, we calculated the preference index (PI) and the depth of the preference index (dPI). The PI is a measure of the selectivity of the response that takes into account the magnitude of the neuronal activity for each LED position. It was computed as defined by Moody and Zipser [54]: *PI* = (*n*- ∑*_i_r_i_/r_pref_*)/(*n-1*), where *n* is the number of positions, *r_i_* the activity for position *i*, and *r_pref_* the activity for the preferred position. The PI value ranges between 0 and 1. A value near 0 indicates the same magnitude of response for all positions, whereas a value near 1 indicates a strong preference for one position. The dPI was computed by the formula: *dPI* = (*r_pref_*−*r_nonpref_*)/(*r_pref_*+*r_nonpref_*), where *r_nonpref_* is the activity in the non preferred position. The dPI index, also ranging from 0 to 1, is a measure of the strength of modulation of the neural activity induced by the change of fixation depth. The difference between index distribution in different epochs was assessed with a Kolmongorov-Smirnov test (two-tailed, P<0.05) and the comparison of indexes in the same cell with the Wilcoxon signed-rank test (P<0.05).

In the ANOVA significant cells a spike density function (SDF) was calculated (Gaussian kernel, halfwidth 20 ms) for each trial and then averaged over all the trials of a given LED position. We found the maximum discharge frequency of the neuron among the behavioral epochs of interest and used it to normalize SDFs. Population SDFs were constructed separately for every epoch of activity by averaging the individual SDFs of all the cells modulated in that epoch [55]. We computed both the population SDFs of the five LEDs in each row separately and the average of all rows. In all cases, the curves of the five positions were statistically compared with a permutation test with 10,000 iterations comparing the sum of squared errors of the actual and randomly permutated data (pairwise fixed interval). The intervals of the curve comparisons were: a) from 50 ms before saccade onset till 100 ms after it for the neurons modulated in perisaccadic epoch and b) from 100 ms after saccade onset till 1100 ms after it for the neurons modulated in fixation epoch. Taking into account that the average duration of the saccades was about 50 ms, the duration of the permutation interval for the perisaccadic cells was set to be roughly equal to the duration of the corresponding epoch. This criterion was also applied for the cells modulated in fixation. Considering that the duration of the fixation epoch was 1 s or 1.5 s, comparisons of the corresponding SDFs were performed in time intervals from 100 ms to 1100 ms after saccade onset. To find the latency at which two curves started to differ significantly from each other a pairwise permutation test was performed using a 100 ms time window that slid along the curves in 20 ms increments starting from 500 ms before the behavioral event used for the SDF alignment (pairwise sliding permutation test). The latency was defined as the midpoint of the first of three consecutive bins in which the permutation test was significant (P<0.05).

We also performed a two way ANOVA test to quantify the relative influence of both fixation depth and the part of space fixated. In each data sample we applied a Kolmogorov-Smirnov test of normality (two-tailed, P<0.05). To compare groups we used an ANOVA or Kruskal-Wallis test, according to dataset normality. For all statistical tests P<0.05 was the significance level.

A multiple linear regression analysis was used to relate the activity in each epoch to three saccadic amplitude parameters: the vector of vergence and version change (delta vergence and delta version, respectively) and the amplitude of version change (delta version amplitude). The regression coefficients were calculated with the stepwise procedure and then were normalized. The standardized coefficients allowed a comparison of the influence of the independent variables.

All analyses were performed using custom scripts written in MATLAB (Mathworks, Natick, MA, USA).
